# RNA-Seq Analysis Reveals the Molecular Mechanisms Regulating the Development of Different Adipose Tissues in Broiler Chicks

**DOI:** 10.3390/ani14060899

**Published:** 2024-03-14

**Authors:** Shuo Wei, Xincheng Kang, Felix Kwame Amevor, Xiaxia Du, Youhao Wu, Zhengyu Xu, Xueqing Cao, Gang Shu, Xiaoling Zhao

**Affiliations:** 1 State Key Laboratory of Swine and Poultry Breeding Industry, College of Animal Science and Technology, Sichuan Agricultural University, Chengdu 611130, China; 13541399198@163.com (S.W.); kangxincheng@stu.sicau.edu.cn (X.K.); amevorfelix@gmail.com (F.K.A.); 15680811065@163.com (X.D.); wuyouhao2021302122@163.com (Y.W.); 18791322009@163.com (Z.X.); 13835890389@163.com (X.C.); 2 Farm Animal Genetic Resources Exploration and Innovation Key Laboratory of Sichuan Province, College of Animal Science and Technology, Sichuan Agricultural University, Chengdu 611130, China; 3 Key Laboratory of Livestock and Poultry Multi-omics, Ministry of Agriculture and Rural Affairs, Sichuan Agricultural University, Chengdu 611130, China; 4 Department of Basic Veterinary Medicine, Sichuan Agricultural University, Chengdu 611130, China; dyysg2005@sicau.edu.cn

**Keywords:** broiler chick, adipose tissue, RNA-seq, differential gene, weighted gene co-expression network analysis

## Abstract

**Simple Summary:**

The genetic basis underlying the development of different adipose tissues in broiler chicks requires extensive study. The molecular mechanisms and specific transcripts associated with fat development and deposition remain unknown. However, little is known about the relationship between the changes in the expression of genes and fat development and deposition in chickens. Thus, the molecular mechanisms regulating the development of different adipose tissues may differ. Hence, studying the mechanisms regulating the growth and development of different adipose tissues during the early stages of broiler development is important in poultry breeding. Therefore, in this study, RNA-seq analysis was utilized to reveal the molecular mechanisms regulating the development and deposition of different adipose tissues in broiler chicks. The results obtained showed that genes such as *MYOG*, *S100A9*, *CIDEC*, *THRSP*, *CXCL13*, and *NMU* were related to the growth and development of adipose tissue. Further, genes such as *HOXC9*, *AGT*, *TMEM182*, *ANGPTL3*, *CRP*, and *DSG2* were identified to be associated with the distribution of adipose tissue, and *MN1*, *ANK2*, and *CAP2* were related to the growth of adipocytes. Therefore, the candidate genes identified in this study could be used in chicken selection to increase or decrease specific adipose tissues in broiler chickens.

**Abstract:**

In an effort to enhance growth rates, chicken breeders have undertaken intensive genetic selection. In the selection process, the primary aim is to accelerate growth, inadvertently leading to new chicken breeds having an increased capacity for rapid adipose tissue accumulation. However, little is known about the relationship between changes in gene expression and adipose tissue accumulation and deposition in chickens. Therefore, in this study, RNA-seq analysis was utilized, and transcriptome data were obtained from the abdominal fat, thoracic subcutaneous fat, and clavicular fat on day 1 (d1), day 4, day 7, day 11, and day 15 to reveal the molecular mechanisms regulating the development and deposition of different adipose tissues in broiler chicks. The results showed that the key period for adipocyte differentiation and proliferation was between d4 and d7 (abdominal fat development) and between d1 and d4 (chest subcutaneous fat and clavicular fat). In addition, candidate genes such as *MYOG*, *S100A9*, *CIDEC*, *THRSP*, *CXCL13,* and *NMU* related to adipose tissue growth and development were identified. Further, genes (*HOXC9*, *AGT*, *TMEM182*, *ANGPTL3*, *CRP*, and *DSG2*) associated with the distribution of adipose tissue were identified, and genes (*MN1*, *ANK2*, and *CAP2*) related to adipose tissue growth were also identified. Taken together, the results from this study provide the basis for future studies on the mechanisms regulating adipose tissue development in chickens. Further, the candidate genes identified could be used in the selection process.

## 1. Introduction

Chickens provide an inexpensive dietary protein source for human consumption, and they also serve as an important animal model for scientific research [1,2,3,4]. Due to intensive genetic selection, broiler chickens accumulate adipose tissue rapidly for a high growth rate [1,2,5]. In addition, during fetal development, adipose tissue is formed from the differentiation of mesenchymal stem cells into adipocytes. Then, the adipocyte differentiates into two phases, namely the determination stage and the terminal differentiation stage [6]. The differentiation and proliferation of adipocytes mainly occur at the embryonic and young stages of chicken development. There is no adipocyte differentiation during the early embryonic developmental stage; however, the adipocytes begin to differentiate from bone marrow-derived mesenchymal stem cells at the late stage of embryonic development prior to hatching. Thereafter, they differentiate into preadipocytes and then gradually change to mature adipocytes [6]. The rate of adipocyte proliferation is slow in broiler chicks; however, triglyceride accumulation is high in mature broilers, thereby increasing adipocyte volume [7]. This regulates the function and metabolism of the existing adipocytes, as well as regulates energy metabolism and endocrine function [8]. The juvenile stage is the critical period for fat development in chicks; therefore, it is important to study early fat development in chickens to understand the processes involved in regulating adipocyte proliferation and differentiation, as well as to explore the mechanisms regulating lipid metabolism in chickens. Chicken breeders select chickens based on their fat accumulation, because excessive accumulation is a health and economic concern for the poultry industry and regular consumers [5].

Domestic broiler chickens are used for studying the mechanisms regulating adipocyte hyperplasia during their development. During the first week post-hatch, chicken adipose tissue grows and develops rapidly via the adipocyte hyperplasia, compared with hypertrophy. Therefore, an increase in the number of adipocytes at the early stage of development is a common phenotypic characteristic of some chicken breeds selected genetically for excess adiposity [1,2]. Studies have reported that the development of the adipocytes of both mammals and chickens is regulated by a series of molecular factors, such as the activation of peroxisome proliferator-activated receptor gamma (*PPARγ*) and CCAAT-enhancer-binding protein alpha (*CEBPα*) [9].

There is a range of genetic and nongenetic factors such as breed, sex, nutrition, age, environment influence, and fat deposition in broiler chickens [10,11,12]. Fats can be deposited at different parts of the chicken body; however, there are differences in adipose tissue development at the different body sites of chickens [13]. Fat in the chest, abdomen, neck, and other parts of the body play different functions, such as thermogenesis and energy storage [14]; however, there may be differences in the mechanisms regulating this process. Several factors such as nutrition, genetics, and age affect fat deposition in poultry. However, due to the interaction of multiple factors, the existence of different adipose tissues, the regulatory mechanisms, and the law of fat deposition in chicks have not been effectively explored. In order to perfectly predict the law of fat deposition in poultry, it is important to conduct an in-depth study through transcriptome sequencing to explore the molecular mechanisms regulating this process.

Previous studies have reported that fat development mainly focuses on the comparison between different chicken breeds or different fat content models, such as comparing the differences between high- and low-fat broiler chickens to determine the main genes regulating fat development [15,16], as well as exploring the mechanisms regulating adipocyte proliferation and differentiation from a single gene level [17,18,19,20]. Fat accumulation varies with different developmental stages in chickens. For instance, fat accumulation begins in the muscle and then further accumulates in the abdomen during embryonic development; however, during the fast-growing period, the opposite trend is reported. Fat development also depends on the amount of lipids synthesized and stored by the adipocyte [21]. Understanding the mechanisms regulating fat development and accumulation is complicated; however, there exists a correlation between the development of adipocyte and the expression of candidate genes in preadipocytes, for instance, genes that are involved in transcription regulation and the differentiation of adipocytes and lipid metabolism [21]. Transcription factors such as PPARγ, C/EBPs, and ADD1 (SREBP1) regulate adipocyte differentiation, whereas genes such as *aP2*, *PEPCK*, and *SCD1* play vital roles in adipocyte differentiation [22,23].

Several researchers used transcriptome sequencing to study the molecular mechanisms and important pathways regulating several biological processes in animals [24,25,26]. Adipogenesis is a multi-step process controlled by several enhancers and inhibitors [27]. Fat deposition is regulated by several tissues; however, the contribution of these tissues changes significantly during the developmental processes. Therefore, the aim of this study was to focus on different developmental periods and the related transcriptomic changes of abdominal fat (Ab), thoracic subcutaneous fat (Br), and clavicular fat (Cl) tissues to reveal the molecular mechanisms and significant pathways regulating chicken adipose tissue development.

## 2. Materials and Methods

All animal experimental procedures were approved by the Institutional Animal Care and Use Committee of Sichuan Agricultural University (approval number: SYXK2019-187). All the experiments were conducted according to the rules provided by the Sichuan Agricultural University Laboratory Animal Welfare and Ethics Committee.

### 2.1. Experimental Animals, Design, and Sample Collection

A total of 300 fertilized eggs obtained from Tianfu broiler breeder chickens were incubated at the Poultry Breeding Unit of Sichuan Agricultural University, China. During the incubation, the unfertilized eggs and dead embryos were selected, recorded and discarded on the 5th and 18th days of incubation, respectively. After hatching, a total of 120 healthy male chicks were allocated into three groups, consisting of 40 chicks each. These experimental groups were established to facilitate the collection of three distinct types of adipose tissues, namely Ab, Br, and Cl, from each replicate. Standard feeding reference (DB51/T 2820-2021) was followed, and a 24 h light program was provided. In the first week, the temperature was set at 34–36 °C; then, in the second week, the temperature was set at 33–35 °C. Throughout the experiment, feed and water were provided ad libitum. The basal diet and nutritional levels are shown in the Supplementary Table S1.

Tissue samples were collected on day 1 (d1), day 4 (d4), day 7 (d7), day 11 (d11), and day 15 (d15). The chicks were sacrificed by oral bleeding after 12 h of fasting, and then the skin was quickly and carefully peeled off. According to the distribution of the adipose tissue in the Supplementary Figure S1, the Ab, Br, and Cl were collected, weighed, and recorded, and then some parts were stored in 4% paraformaldehyde. Thereafter, the other samples were transferred to a −80 °C chamber and subsequently stored for sequencing and validation.

### 2.2. Experimental Animals’ Histological and Morphological Observation

The tissues were fixed for 24 h and embedded in paraffin, and then the tissue sections were stained with hematoxylin and eosin (HE) for histological and morphological observation. The adipocyte area, adipocyte density, and adipocyte diameter of each section were then measured using Image pro plus 5.0 (Media Cybernetics, Rockville, MD, USA).

### 2.3. Biochemical Analysis

The fat tissue samples were inserted into an absolute ethanol solution. They were ground and homogenized with a tissue grinding rod, and centrifuged at 3000 rpm for about 10 min at 4 °C to obtain the supernatant for biochemical analysis. Then, the triglyceride levels in the adipose tissue were determined using the triglyceride kit (Nanjing Jiancheng Bioengineering Institute, Nanjing, China) according to the manufacturer’s instructions.

### 2.4. Gene Library Construction and Quality Control

Total RNA (≥1 μg) was used as the starting RNA, and a gene library was constructed using the NEBNext^®^ UltraTM RNA Library Prep Kit (Illumina, San Diego, CA, USA). The PCR products were purified using the AMPure XP Beads (Beckman Coulter, Pasadena, CA, USA), and then were quantified using fluorescence spectroscopy (Qubit 3.0 Fluorometer) (Waltham, MA, USA). Thereafter, they were diluted to 1.5 ng/μL. Finally, quality check was performed for the inserted sequences from the library using an Agilent 2100 bioanalyzer (Agilent Technologies, Palo Alto, CA, USA). After the library construction was completed, the NovaSeq6000 (Illumina, San Diego, CA, USA) platform was used for two-end sequencing, and the sequencing length was 150 bp. The transcriptome sequencing of this experiment was completed by Beijing Novogene Biotech Co., LTD (Beijing, China).

### 2.5. Differential Expression Gene Analysis

Cutadapt was used to remove the low-quality data, and then HISAT2v2.0.5 (Texas A&M University, Baltimore, MD, USA) was used to align the clean reads with the reference genes [28]. The correlation coefficient between the samples was calculated and the principal component analysis diagram was drawn. The reference genome sequence used in this study was the chicken reference genome sequence with version number GCF_000002315.6_GRCg6a in NCBI database. Supplementary Table S3 shows that the total comparison rate was approximately 90%, indicating that the data were valid. The R DESeq2 (Cornell University, Ithaca, NY, USA) was used for the identification of differentially expressed genes (DEGs) [29]. When |log2foldchange| ≥ 1 and *p* < 0.05, genes were considered DEGs.

### 2.6. Functional Enrichment Analysis

ClusterProfiler (3.4.4) software was used for Gene Ontology (GO) and Kyoto Encyclopedia of Genes and Genomes (KEGGs) enrichment analysis of DEGs [30]. For GO functional enrichment and KEGGs pathways, the *p* ≤ 0.05 was used as the threshold of difference in significance.

### 2.7. RNA Extraction and Real-Time Quantitative PCR

The adipose tissue samples were ground into powder using an automatic tissue sample grinder (Chengdu Shunhe, Chengdu, China), and the total RNA was extracted using TRIzol reagent (Takara, Dalian, China) according to the manufacturer’s instructions [30]. For each of the three different adipose tissues (abdominal fat, thoracic subcutaneous fat, and clavicular fat) at every developmental time point (day 1, day 4, day 7, day 11, and day 15), three samples were collected and ground. The concentration and purity of the extracted RNA were determined using the Nanodrop 2000 C (Thermo Fisher Scientific, Waltham, MA, USA) at an absorbance ratio of A260/280. Single-stranded cDNA was synthesized using PrimeScript RT Reagent Kit (Takara, Dalian, China). Subsequently, the TB Green^®^ Premix Ex Taq™ (Takara, Beijing, China) was used for the qRT-PCR analysis according to the manufacturer’s instructions, and the 2^−ΔΔCt^ method [31] was used to calculate the fold changes in the gene expression. The primers used for the qRT-PCR are listed in the Supplementary Table S2.

### 2.8. Weighted Gene Co-Expression Network Analysis

Weighted Gene Co-expression Network Analysis (WGCNA) was performed using the WGCNA package v.1 [32] with the default settings and minor modifications. Set minModuleSize to 30 and mergeCutHeight to 0.75. The soft threshold function was used for the network topology analysis to calculate the soft threshold β. The number of weight parameter β was selected as 20, the R2 (the square of correlation coefficient) was selected as 0.8, the soft threshold β was 6, and the average connectivity between the genes was approximately 250, which met the requirements of module division (Supplementary Figure S2). The topological overlap matrix (TOM) was constructed, and a dendrogram was constructed by module detection and by merging related modules to calculate the correlation of module–trait relationships.

### 2.9. Identification of Gene Hub

When the Gene Significance (GS) was greater than 0.2 and the Module Membership (MM) was greater than 0.8 and less than 0.05, the most closely connected top 10 genes were classified as hub genes according to the module size. Then, the Cytoscape v.3 was used for the gene interaction network mapping.

### 2.10. Statistical Analysis

All data were analyzed by one-way analysis of variance (ANOVA) using SPSS version 25.0 software (International Business Machines Corporation, Armonk, NY, USA). The results were expressed as the mean ± mean SD, and a significant difference was indicated at *p* < 0.05.

## 3. Results

### 3.1. Differences in the Phenotype of the Adipose Tissues

**Figure 1:**
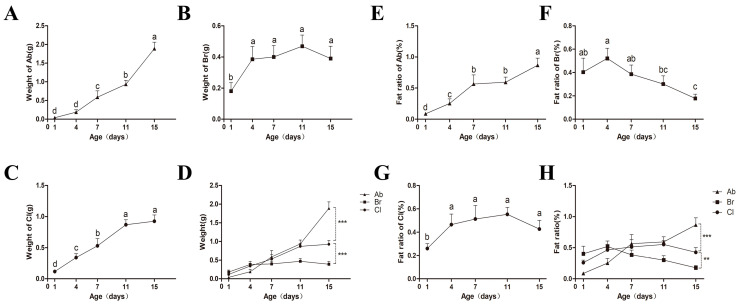
Weight and ratio of the Ab, Br, and Cl tissues at different time points. Values without the same letter differed significantly (*p* < 0.05). (**A**–**C**) Weight of the Ab, Br, and Cl. (**D**) Comparison of Ab, Br, Cl tissues’ fat weights. Significant differences were observed between the Ab and Cl, and the Cl and Br (*p* < 0.001). (**E**–**G**) The ratio of the Ab, Br, and Cl tissues. (**H**) Comparison among the fat ratio of the Ab, Br, and Cl. Significant differences were observed between the Ab and Cl (*p* < 0.001), and Cl and Br (*p* < 0.01), respectively. The ** and *** indicated moderate and strong correlation, respectfully.

The weight of the abdominal fat (Ab), thoracic subcutaneous fat (Br), and clavicular fat (Cl) tissues at different developmental periods were measured and recorded (Figure 1A–C), and the results showed that the weight of the adipose tissues of each body part showed an increasing trend with increasing age. The weight of the Ab increased significantly after d4 and reached the highest point on d15. There was no significant difference observed in the weight of Br on d4, d7, d11, and d15 (*p* > 0.5); however, it was significantly higher compared to d1 (*p* < 0.5). The weight of Cl was significantly higher at d15 than at d1, d4, and d7, with no significant difference between d11 and d15. On d15 (Figure 1D), the weight of the Ab fat was significantly higher than that of Cl (*p* < 0.001); however, the weight of Cl was significantly higher than that of Br (*p* < 0.001).

Based on the observed body weights of the experimental chickens (Table S4), the fat percentage of the three adipose tissues (Figure 1E–G) were calculated, and the results showed that the fat percentage of the Ab increased with increasing age and reached the highest value on d15. It was observed that the fat percentage of Br reached the highest on d4, and then showed a downward trend, and later showed a significantly lower trend on d15 as compared to on d1, d4, and d7 (*p* < 0.5). Further, the fat percentage of Cl increased significantly at first and then decreased from d1 to d15, with a lower fat percentage on d1 compared with the other time points. On d15 (Figure 1H), the fat percentage of Ab was significantly higher than that of Cl (*p* < 0.001), whereas the fat percentage of Cl was significantly higher than that of Br (*p* < 0.01).

The weights of all the adipose tissues measured increased with increasing age, and significant differences were observed among the developmental time points between the adipose tissues, with the Ab developing later than the Br and Cl between d1 and d15. Further, the growth intensity of the Ab was the strongest, followed by the Cl and then the Br. The deposition rate of the Ab was higher than that of the body weight, and the deposition rate of the Br was lower than the body weight, whereas the deposition rate of Cl was comparable to the body weight.

### 3.2. Differences in Adipocytes Morphology among the Ab, Br, and Cl

**Figure 2:**
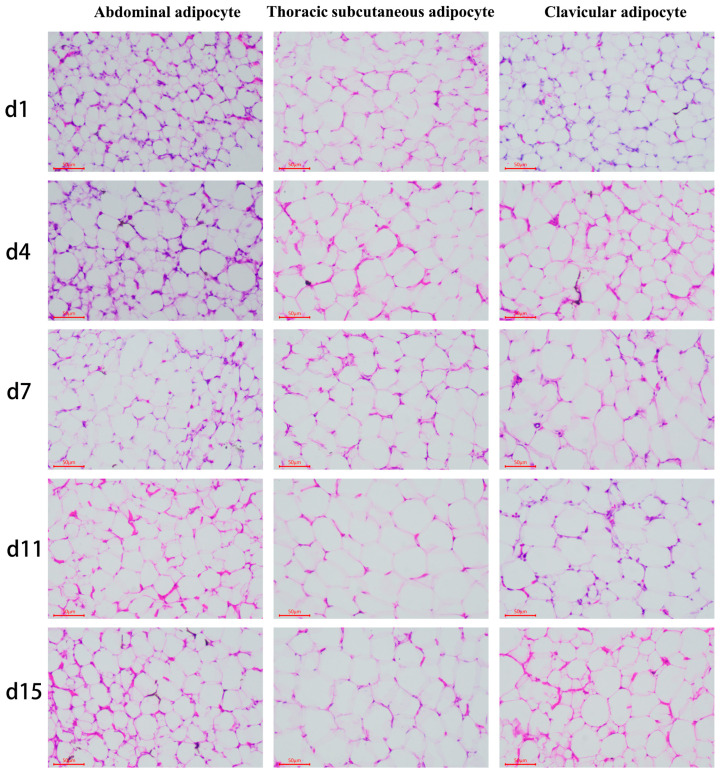
Morphological observation of the various adipose tissues (50 μm).

**Figure 3:**
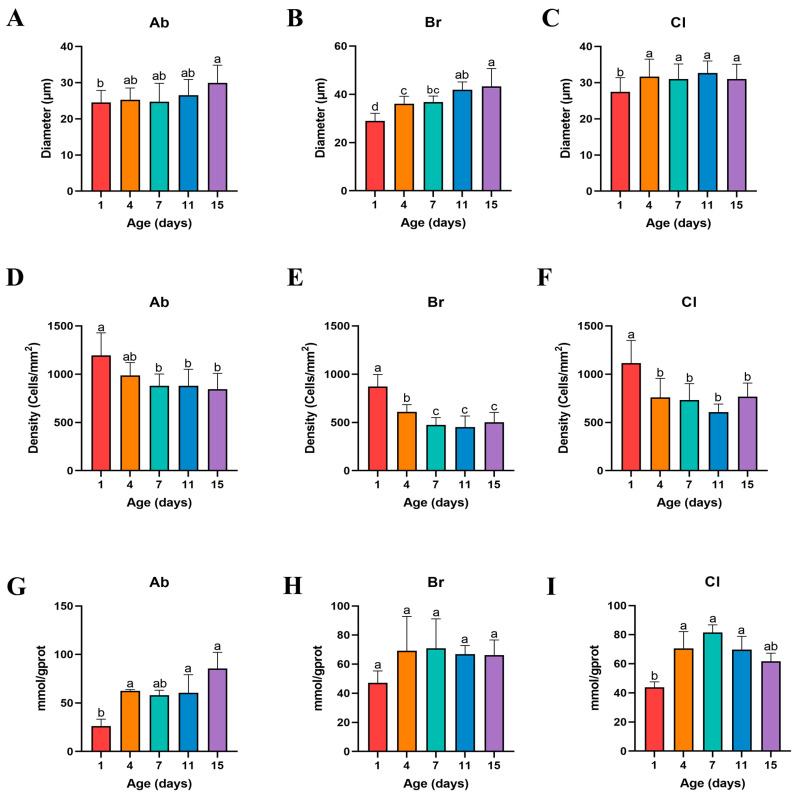
The levels of triglyceride in the Ab, Br, and Cl, and the cell density and diameter of each adipose tissue section. Bars without the same letter differed significantly (*p* < 0.05). (**A**–**C**) Adipocyte diameters in the Ab, Br, and Cl tissues. (**D**–**F**) Adipose cell density in the Ab, Br, and Cl tissues. (**G**–**I**) Triglyceride levels in the Ab, Br, and Cl.

The morphological examination of the fat tissue samples was determined (Figure 2), and the cell (adipocyte) density and cell diameter were counted, and the results showed that the adipocyte of Ab, Br, and Cl tissues were progressively larger in diameter (Figure 3A–C). The adipocyte diameter of Ab on d15 was significantly higher than that on d1 (*p* < 0.05); however, there was no significant difference between the adjacent time points (*p* > 0.05). The diameter of the Br adipocytes was significantly higher on d4 than that on d1 (*p* < 0.05); however, it was significantly higher on d15 as compared to d1, d4, and d7 (*p* < 0.05). There was no significant difference observed between d4 and d7, and d7 and d11 (*p* > 0.05), whereas the diameter of the adipocyte of Cl on d1 was significantly lower than that on d4, d7, d11, and d15 (*p* < 0.05). Compared with the Ab and Cl, the adipocyte diameter of the Br varied greatly during the early development.

The fat density of each of the adipose tissue showed a decreased trend with age (Figure 3D–F). The density of the adipocyte of the Ab on d1 was significantly higher than that on d4, d7, d11, and d15 (*p* < 0.05), whereas the density of the adipocytes in the Br was significantly higher on d1 than on d4 (*p* < 0.05), but was significantly higher on d4 than that on d7, d11, and d15 (*p* < 0.05). The density of the Cl adipocyte was significantly lower on d4, d7, d11, and d15 than that on d1 (*p* < 0.05). The density of the adipocytes reflects the number and the degree of adipocyte accumulation in the adipose tissue of the broiler chicks. In this study, the Ab tissue has the largest degree of fat accumulation, followed by Cl tissue, whereas the degree of fat accumulation of the Br tissue was the smallest, which was consistent with the results presented in Figure 1.

The level of triglyceride (TG) (Figure 3G–I) in the Ab tissues on d1 was lower than that recorded on d4, d7, d11, and d15 (*p* < 0.05), and was the highest on d15. Further, the level of TG content in the Br tissue was lower on d1 than on d4; however, there were no significant differences observed (*p* > 0.05). On day 1, the level of TG in the Cl tissue was low as compared to that on d4, d7, and d11 (*p* < 0.05), whereas the level of TG was highest on d7 compared to that on d1 (*p* < 0.05).

The diameter of the adipocytes of the Ab, Br, and Cl tissues was the smallest on d1, whereas on d1, the adipocyte diameters of the Br and Cl tissues were significantly smaller than that on d4 (*p* < 0.05). The TG levels in all the three adipose tissues were lowest on d1, which was consistent with the diameter of each of the adipose tissues. The level of TG in the Ab on d15 was higher as compared with the Br tissue on d4, whereas its level in the Cl tissue was highest on d7. Hence, the proliferation and differentiation of Ab tissue was higher than that of the Cl and Br tissues with increasing age.

### 3.3. Transcriptome Sequencing and DEGs Analysis

**Figure 4:**
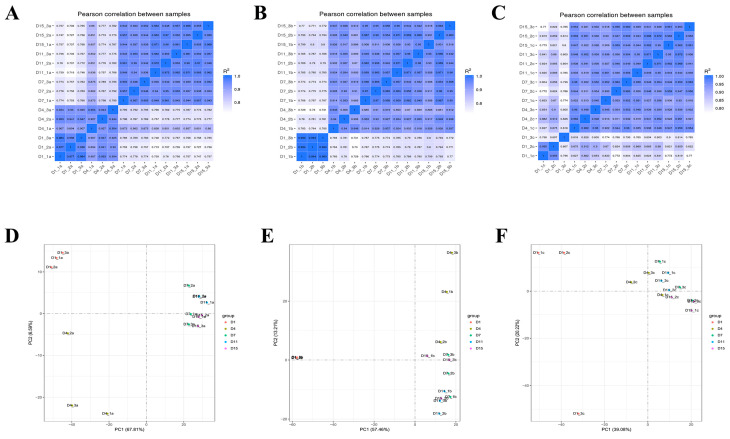
Heat maps of the inter-sample correlations, and PCA results for the Ab, Br, and Cl tissues. (**A**–**C**) Heat map of the correlation in the Ab (**A**), Br (**B**), and Cl (**C**). (**D**–**F**) Principal component analysis (PCA) results of Ab (**D**), Br (**E**), and Cl (**F**) tissues.

We found that the Cl in the correlation analysis of the (D1_3c) samples and (D1_1c), (D1_2c) was due to the poor repeatability (Figure 4C); hence, these samples were not used in the analysis of the differences between the groups.

**Figure 5:**
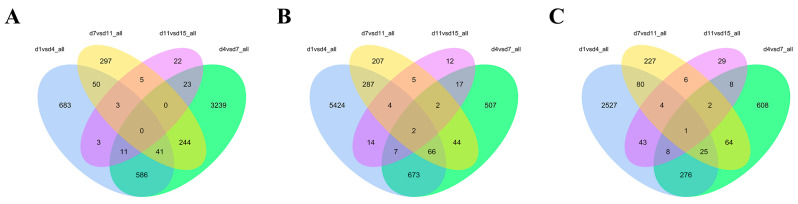
Venn diagram of the differentially expressed genes in the Ab (**A**), Br (**B**), and Cl (**C**) tissues.

**Figure 6:**
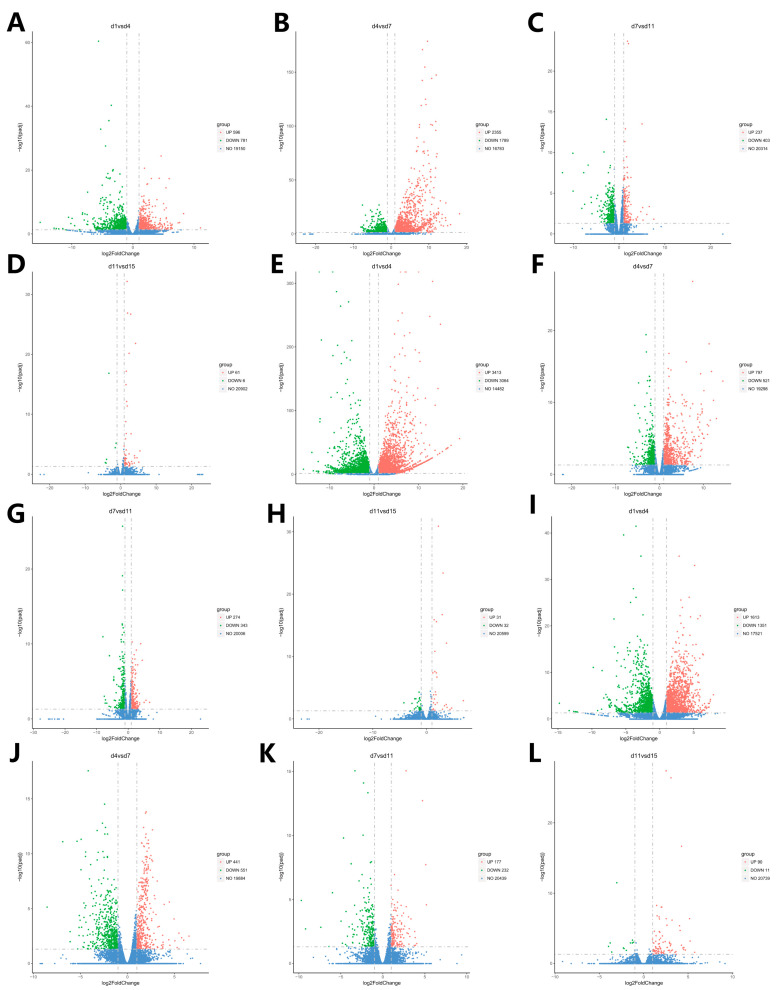
Volcano plot of the distribution of the DEGs at the adjacent time points in the Ab, Br, and Cl tissues. (**A**–**D**) Volcanic map of DEGs in the Ab. (**E**–**H**) Volcanic map of DEGs in the Br. (**I**–**L**) Volcanic map of DEGs in the Cl. Note: The abscissa is log2fold change, the ordinate is −log10 *p*-value, and the blue dotted line represents the threshold line of the differential gene screening criteria.

A total of 6228 DEGs were screened in the Ab tissues. Among them, 1377 were identified on d1 vs. d4, whereas 4144 were identified on d4 vs. d7; 640 were found on d7 vs. d11; and 67 were identified on d11 vs. d15. In the Br tissue, a total of 8475 DEGs were screened, among which 6477 were identified on d1 vs. d4, 1318 were identified on d4 vs. d7, 617 were detected on d7 vs. d11, and 63 were found on d11 vs. d15. In addition, a total of 4466 DEGs were screened in the Cl tissues, among which 2964 were identified on d1 vs. d4, 992 were found on d4 vs. d7, 409 identified on d7 vs. d11, and 101 were found on d11 vs. d15. The Venn intersection was performed for the differential genes (Figure 5). The distribution of the number of differentially expressed genes between the adjacent time points in the same adipose tissue is presented in Figure 6.

**Figure 7:**
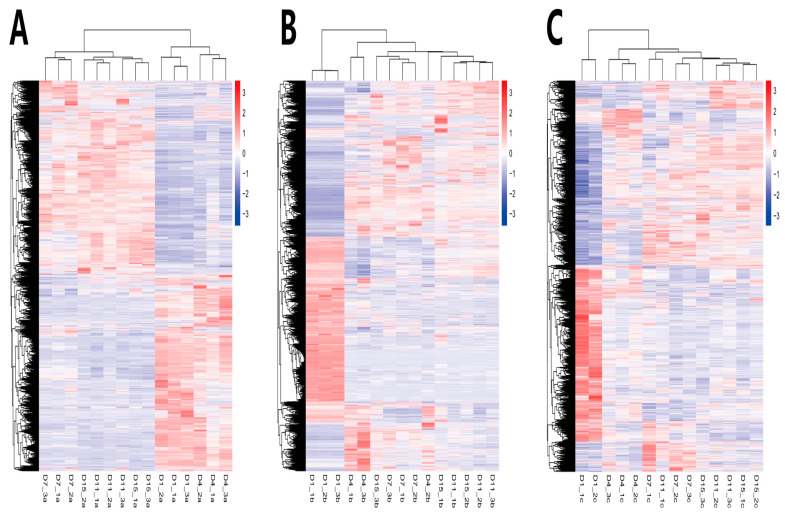
DEGs heatmap in the Ab, Br, and Cl. (**A**) Ab. (**B**) Br. (**C**) Cl. Note: The abscissa is the sample name, and the ordinate is the normalized value of fpkm.

In order to compare the DEGs between the different experimental groups, the DEGs were clustered with similar expression patterns (Figure 7). Based on the expression patterns of the DEGs, the growth and development of the Ab tissue changed significantly from d4 to d7, whereas the Br and Cl tissues changed significantly from d1 to d4. Therefore, the Ab tissue was selected on d4 and d7, and the Br tissue was selected on d1 and d4, whereas the Cl tissue was selected on d1 and d4 for subsequent enrichment analysis.

### 3.4. GO and KEGGs Pathway Enrichment Analysis

GO enrichment analysis was performed on the Ab tissue on d4 and d7, Br tissue on d1 and d4, and Cl tissue on d1 and d4. The top 10 most significant terms were selected in Molecular Function (MF), Cellular Component (CC), and Biological Process (BP), respectively.

**Figure 8:**
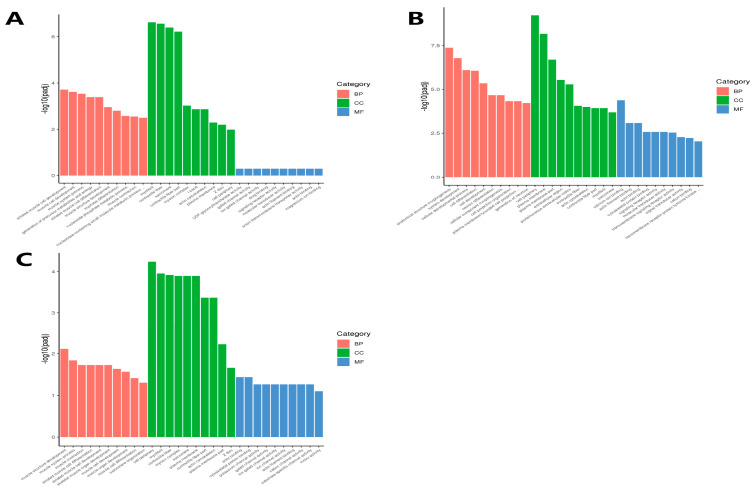
GO enrichment analysis of the differential genes in the Ab, Br, and Cl tissues. GO, Gene Ontology. (**A**) Ab, (**B**) Br, (**C**) Cl. Note: The abscissa is the GO term, and the ordinate is the significance level of the GO term enrichment.

The DEGs in the Ab tissues were mainly enriched in the precursor metabolites and energy production, nucleoside phosphate metabolism and muscle cell differentiation in BP, and were mainly enriched in cell periphery and plasma membrane in CC, but were not significantly enriched in MF. Moreover, most of these significantly enriched differentially expressed genes were up-regulated on d7 (Figure 8A). The DEGs in the Br tissues were mainly enriched in the cell differentiation, cell development, and cell component morphogenesis in BP, plasma membrane and cell periphery in CC, and calcium ion binding and cytoskeletal protein binding in MF (Figure 8B). The DEGs obtained from the Cl tissue were mainly enriched in the muscle-related functions such as muscle contraction and muscle cell differentiation in BP, cell periphery and plasma membrane parts in CC, and actin and cytoskeletal protein binding in MF (Figure 8C).

Ab tissue was selected on d4 and d7, whereas Br tissue was selected on d1 and d4, and the Cl tissue was selected on d1 and d4 for KEGGs enrichment analysis, and the top 20 most significant KEGGs pathways were selected.

**Figure 9:**
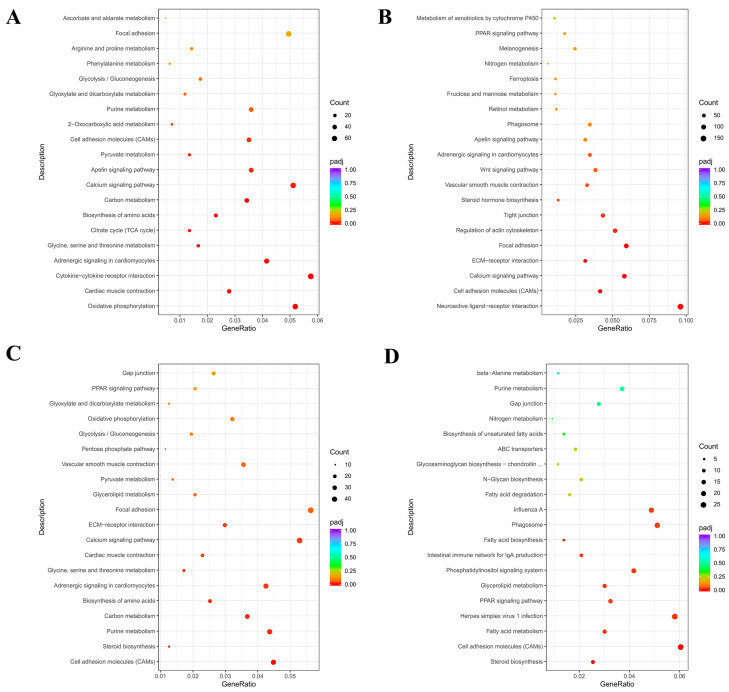
KEGGs enrichment analysis of the differential genes in the Ab, Br, and Cl tissues. KEGGs. (**A**) Ab, (**B**) Br, (**C**) Cl, (**D**) KEGGs enrichment pathway map of the down-regulated differentially expressed genes in the Cl. Note: The abscissa is the ratio of the number of differential genes annotated to the KEGGs pathway to the total number of differential genes, and the ordinate is KEGGs pathway.

DEGs in the Ab tissues were mainly enriched in carbon metabolism, citric acid cycle (TCA cycle), and apelin signaling pathway (Figure 9A). The growth and development of adipose tissue involve numerous oxidative phosphorylation reactions in the mitochondria. Fatty acids produced by lipolysis participate in the TCA cycle, and apelin is an adipokine secreted by the adipocytes, which are related to adipose differentiation. DEGs in the Br tissues were mainly enriched in fat-related pathways such as Extracellular Matrix (ECM) receptor signaling pathway, PPAR signaling pathway, and cell adhesion molecules (CAMs) (Figure 9B). A total of 28 genes were enriched in the PPAR pathway, and 24 of them were significantly down-regulated. DEGs in the Cl tissues were mainly enriched in pathways such as CAMs and steroid biosynthesis (Figure 9C). Adipose tissue regulates fat metabolism by secreting cell adhesion molecules. Interestingly, almost all of the differential genes related to fat-related pathways (enriched by KEGGs) were down-regulated. Further enrichment analysis of the down-regulated genes (Figure 9D) was performed, and we found that these DEGs were significantly enriched in the PPAR, fatty acid metabolism, and fatty acid biosynthesis pathways.

### 3.5. Validation of Sequencing Data by RT-qPCR

**Figure 10:**
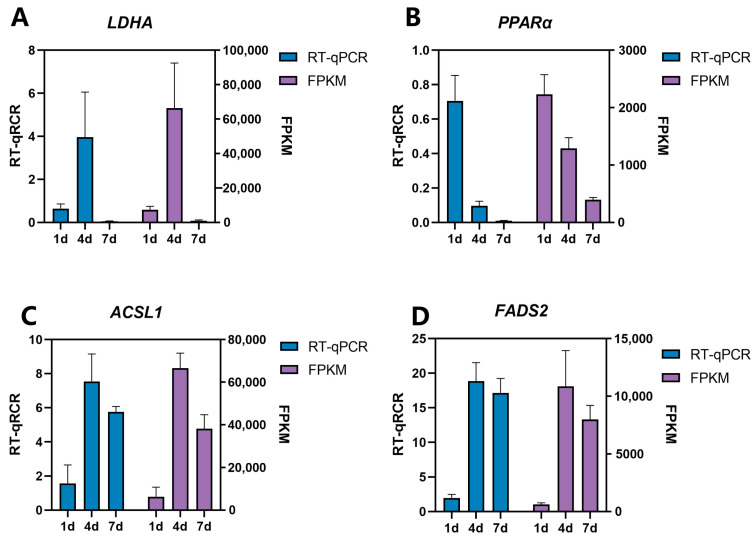
RT-qPCR verification of differential genes. Ab tissue samples (d1, d4, and d7) were selected to identify LDHA and PPARα expression, and Cl tissue sample (d1, d4, and d7) was selected to identify the expression of ACSL1 and FADS2.

In order to verify the accuracy of the transcriptome sequencing results, four genes related to adipose tissue development were randomly selected from the DEGs in the same tissue at different developmental stages, and were subsequently screened by quantitative verification. The quantitative results were consistent with the expression trend of RNA-seq (Figure 10). Therefore, the results of the RNA-seq were accurate and reliable.

### 3.6. WGCNA Module Identification

**Figure 11:**
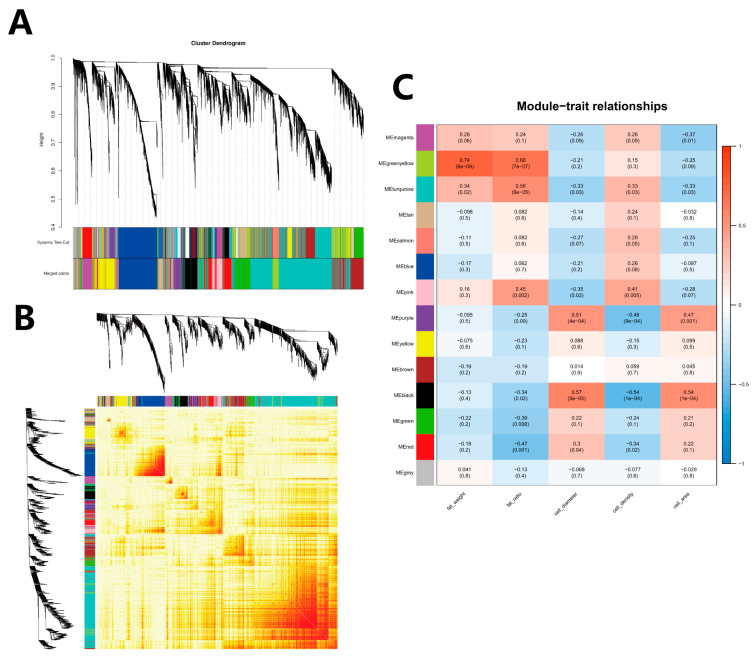
WGCNA and modules–traits relationship. (**A**) WGCNA co-expression module partition result graph. Upper panel: Genes were clustered into different groups. Lower panel: Genes were assigned to modules after dynamic tree cutting and merging. (**B**) Gene network heatmap of the WGCNA module: Each dendrogram represents a module; each branch represents a gene; and the darker the color of each dot (white → yellow → red), the stronger connectivity between the two genes corresponding to the row and column. (**C**) Module and trait correlation heatmap: The vertical axis is the gene module derived from the analysis, the horizontal axis is the trait, the first value at the intersection is the correlation coefficient between the module and the corresponding trait, and the second value is the *p*-value of the coefficient. Values are expressed in scientific notation for clarity and precision, where ‘e’ denotes ‘×10^’. For example, ‘5e-04’ represents 5 × 10^−4^.

Genes with fragments per kilobase of exon model per million mapped fragments (FPKM) less than 1 were removed to construct a gene co-expression network. The dynamic tree cut was used to construct the gene modules. Finally, it was divided into 13 modules (Figure 11A), and the gene clustering heatmap was drawn (Figure 11B).

The correlation between the fat weight, fat percentage, adipocyte diameter, density, and area of each module (Figure 11C) was analyzed. The result revealed that the green-yellow module had the highest positive correlation with the fat weight, and the red module had the highest negative correlation with the fat percentage, and the pink and black modules had the strongest correlation with the adipocyte density. However, the correlation of the adipocyte diameter and area was opposite to that of the adipocyte density, and the red and pink modules were selected for further analysis.

### 3.7. Screening of Hub Genes in WGCNA Module

**Figure 12:**
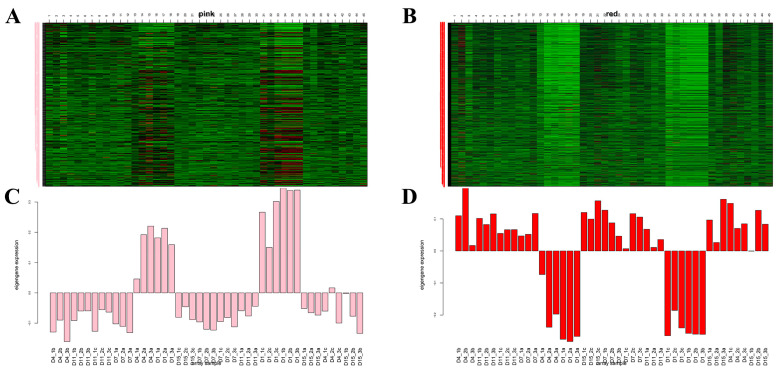
Pink (**A**–**C**) and red (**B**–**D**) modules’ gene expression patterns. (**A**,**B**) The left side of the heat map is the gene name, and the abscissa is the sample name. The corresponding genes that were up-regulated are in red color and those down-regulated are in green. (**C**,**D**) Expression patterns of the module feature values in the different samples; abscissa is the sample name; and the ordinate is the expression level of the gene.

Gene expression patterns of MEpink and MEred modules were plotted (Figure 12A–D).

**Figure 13:**
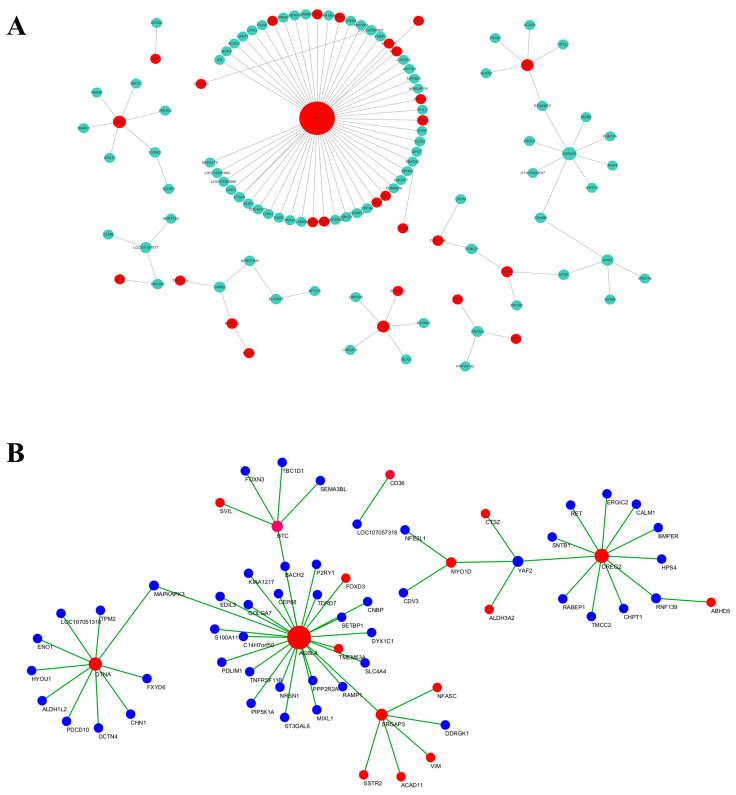
Pink (**A**) and red (**B**) modules’ network interaction diagram.

The genes in the MEpink module were selected, and the top 50 genes with high intramodular connectivity were selected for intersection. Further, a total of 28 genes at the top of the connection were regarded as hub genes. Cytoscape software was used to draw the network diagram (Figure 13A), and MN1, ANK2, and ZSWIM5 were identified as core genes that may have certain roles in the increase in adipocyte volume. According to the gene function enrichment analysis of this module, these genes were mainly enriched in the RNA transport, TGF-β signaling pathway, and MAPK signaling pathway.

The genes in the MEred module were selected, and the top 50 genes with high intramodular connectivity were selected for intersection, with a total of 18 genes. Through the network diagram drawn using the Cytoscape software (Figure 13B), it was observed that core genes such as DTNA, AGBL4, MYO1D, and BTC may have a certain role in the distribution of adipose tissue. Subsequently, the gene function enrichment analysis of this module showed that these genes were mainly enriched in the adipokine signaling pathway, PPAR signaling pathway, and MAPK signaling pathway.

## 4. Discussion

Domestic chicken provides animal protein for humans and can be used as an excellent animal model for scientific studies; however, only a few studies have reported on the regulation of gene expression in chicken adipose tissue. Adipose tissue influences the quality of poultry meat; thus, it plays a significant role in the maintenance of poultry performance [30,33,34]. In addition, fat contents promote meat flavor as well as influences its tenderness [35,36,37]. Adipose tissue is an important source of energy for regulating poultry growth and development. In addition, adipose tissue regulates energy metabolism and feed conversion ratio in poultry, as well as stores energy [38,39]. Studies have reported that adipose tissue plays immunoregulatory roles in poultry. Thus, adipocyte secretes several hormones and cytokines that regulates immune response and inflammatory processes in poultry, thereby maintaining immune homeostasis and promoting disease resistance in poultry [30]. Furthermore, adipose tissue secretes reproductive hormones and metabolic functions to promote sexual maturity and reproduction in animals. Therefore, maintaining proper fat deposition significantly influences hormone balance and reproductive performance in animals [40].

The present study provides new insights into the transcriptome changes in chickens between different developmental stages of Ab, Br, and Cl tissues. In the present study, the results showed that the abdominal fat weight gradually increased with age. Thus, on d15, the abdominal fat weight increased significantly, followed by clavicular fat and then subcutaneous fat. Similarly, on d15, the abdominal fat intensity was the largest, which was followed by the clavicular fat, and subsequently the thoracic fat intensity. Further, fat percentage analysis showed that between d4 and d7, the growth rate of the abdominal fat increased faster than that of the clavicle and thorax fats, indicating the fastest fat deposition rate. This may be attributed to the influence of external environmental factors post-hatch, thereby causing rapid proliferation and differentiation of abdominal fat to maintain body temperature. Previous studies showed that growth and development of fat is influenced by body parts and age [41]. In this study, the results showed that there were different developmental patterns of the three adipose tissues collected on d4 and d14, which was consistent with the results reported in a previous study [42]. In addition, we found in this present study that on d4–d7, the rate of abdominal fat deposition was the fastest, followed by the clavicular fat and then the thoracic subcutaneous fat. This was in agreement with a previous study which reported that abdominal fats grow rapidly post-hatch [43].

In poultry, fat deposition usually focuses on adult chickens, which is mainly caused by increase adipocyte volume [44]. The embryonic and young developmental periods are critical periods for proliferation, differentiation, and development of adipocytes in chickens, which could subsequently influence adipocyte number and fat deposition at the later stages [45,46]. However, there are few studies on the molecular mechanism regulating fat development and deposition in young chicks. In this present study, based on the relevant phenotypic data and transcriptome sequencing data of the Ab, Br, and Cl tissues, we speculated that fat deposition, and the molecular mechanisms regulating this process during the growth and development of these three types of adipose tissues differ; therefore, WGCNA was used to further analyze the differentially expressed genes in the RNA-seq data to help understand the function and biological significance of the DEGs, and to further explore the molecular mechanisms regulating fat deposition in the different adipose tissues. Previous studies have examined differentially expressed genes between the breast muscle and subcutaneous fat in ducks using WGCNA combined with RNA-seq, and they found that these genes were significantly aggregated in multiple pathways related to muscle development and fat deposition, such as MAPK signaling pathway, PPAR signaling pathway, calcium signaling pathway, fat digestion and absorption, and TGF-β signaling pathway. Key genes such as *MSTN, TGFb3, TGFbI,* and *FOXO6* were screened [47]. Similarly, a previous study showed that key genes related to fat deposition such as *GPX1, GBE1, FABP1, ELOVL6,* and *SCD* were screened based on the transcriptome analysis and fat deposition of the intramuscular fat and abdominal fat in broilers [30].

In the present study, the KEGGs signaling pathway analysis showed that DEGs between d4 and d7 in the Ab tissues were mainly enriched in carbon metabolism, TCA cycle and apelin signaling pathway. A study by Heinonen et al. indicated that the growth and development of abdominal fat involves mitochondria oxidative phosphorylation [48]. The present study shows that the differentially expressed genes between d1 and d4 in the Br tissues were mainly enriched in the ECM receptor signaling pathway, PPAR signaling pathway, and CAMs. The differentially expressed genes between d1 and d4 in the Cl tissues were mainly enriched in pathways such as the CAMs and steroid biosynthesis. In addition, we found that Ab and Br were co-enriched in apelin signaling pathway, which is an adipocytokine secreted by adipocytes [49] and it is related to adipocyte differentiation [50]. The Ab and Cl were co-enriched in glycolysis gluconeogenesis, purine metabolism, pyruvate metabolism, and amino acid biosynthesis. This shows that the abdominal and clavicle fats in the broiler chicks have similar processes of energy metabolism during their development. In addition, Br and Cl were enriched in ECM–receptor interaction, PPAR pathway, and CAMs. Studies have shown that adipose tissue regulates fat metabolism by secreting CAMs [51,52]. The ECM–receptor pathway regulates adipocyte development and function by modulating intracellular signaling pathways, forming and maintaining the structure of adipose tissue, and modulating lipid deposition [51,52], whereas the PPAR pathway is associated with regulating lipid metabolism [53,54].

In the present study, it was revealed that the critical period for the abdominal fat development was between d4 and d7, and thus, we found a total of 4144 DEGs, among which 2355 genes were up-regulated and 1789 genes were down-regulated on d7, whereas the top 20 differentially expressed genes were selected from the up-regulated and down-regulated DEGs, respectively. The selected genes, such as MYOG, S100A9, CXCL13, MMP7, and MYF6, were selected as candidate genes affecting fat growth and development. MYOG and MYF6 play important roles in adipocyte proliferation and differentiation [55]. Exogenous PPARγ drives the up-regulation of MYOG expression in vivo. MYOG and MYF6 are regulated by SIRT1 to influence adipocyte proliferation, differentiation, and apoptosis. Exosomes secreted by adipocytes inhibit the expression of MYOG and MYF6 [56]. S100A9 is related to obesity and regulated by P53 to modulate cell cycle and adipogenesis [57]. High-fat feeding promotes the proliferation of preadipocytes by affecting cell cycle, and further causes abdominal fat deposition, which may be related to the enrichment of S100A9 in the pathways related to cell cycle [58]. The CXCL family plays an important role in adipocytes, among which CXCL1 and CXCL16 are chemokines in preadipocytes, and CXCL13 is a chemokine in adipocytes whose expression level is significantly higher than that in the preadipocytes [58].

Further, it was revealed in this study that the critical period for the development of Br tissue was between d1 and d4. After the sequencing analysis of this group, a total of 6477 DEGs were found, among which 3413 were up-regulated and 3064 were down-regulated on d4. The top 20 DEGs were selected from the up-regulated and down-regulated genes, and PKP1, NKX6-1, GC, CIDEC, PLINI, and THRSP genes were found as candidate genes related to the growth and development of thoracic subcutaneous fat on d1–d4. For instance, GC is an important hormone in the hypothalamic–pituitary–adrenal axis. A study showed that the administration of dexamethasone in mice leads to alterations in the expression of BTG1 and CREB1, thereby inducing the whitening of brown adipose tissue and activation of autophagy [59]. CIDEC is a lipid droplet-associated protein that inhibits fat breakdown and further induces accumulation of triglyceride in the adipocytes. Studies have shown that CIDEC is regulated by PPARγ2 [60] and plays an important role in adipocyte differentiation. CIDEC promotes adipocyte differentiation by interacting with AMPKα. AMPK activates the expression of dexamethasone through PPARγ to accumulate lipid droplets in mouse liver [61]. THRSP is also involved in fat metabolism, and THRSP has also been reported as a key gene affecting fat deposition in pig muscle [62]. THRSP gene is associated with liver synthesis and abdominal fat traits in chickens and could be used as a candidate gene for further study.

In addition, the critical period for the growth and development of Cl tissue was between d1 and d4; therefore, from the analysis, a total of 2964 DEGs were found, among which 1613 were up-regulated genes and 1351 were down-regulated genes. The top 20 DEGs from the up-regulated and down-regulated DEGs were selected, respectively. Among them, NMU and SCD were found to be related to adipose tissue development; hence, they were selected as candidate genes. NMU is a polymorphic neurotransmitter that regulates appetite and energy metabolism, and also regulates lipid deposition. In addition, NMU regulates body weight and energy balance in mice [63]. However, NMU knockout rats fed with basal and high-fat diets showed no differences in body weight, fat development, and feed intake. This suggested that NMU may have different functions in different species [64]. Currently, only few studies have validated the role of NMU gene in the growth and development of fat in poultry. SCD is a rate-limiting enzyme that catalyzes the formation of monounsaturated fatty acids into monounsaturated fatty acids, which regulates the synthesis of triglycerides. SCD gene is associated with abdominal fat deposition in chicken [30], and it plays major role in adipocyte formation and fat deposition.

## 5. Conclusions

Taken together, the transcriptomic sequencing result from this study identified several candidate genes (*MYOG*, *S100A9*, *CIDEC*, *THRSP*, *CXCL13*, *NMU*, *HOXC9*, *AGT*, *TMEM182*, *ANGPTL3*, *CRP*, *DSG2*, *DTNA*, *AGBL4*, *MYO1D*, *BTC*, *MN1*, *ANK2,* and *CAP2*) and significant pathways that play important roles in the growth and development of chicken adipose tissue at different developmental stages, as well as revealed critical periods for the proliferation and differentiation of abdominal fat and thoracic subcutaneous and clavicular fats. In addition, the results obtained from this study provide a significant foundation for regulating fat deposition in chicken through future genetic selection or important chicken management practices.

## Data Availability

The data presented in this study are available on request from the corresponding author.

## Supplementary Materials

The following supporting information can be downloaded at: https://www.mdpi.com/article/10.3390/ani14060899/s1, Figure S1: The adipose tissues in different anatomic locations of the chick; Figure S2: WGCNA soft threshold selection; Table S1: The composition and nutritional values of the basal diet (% dry matter); Table S2: Primers used for quantitative real-time PCR (qRT-PCR); Table S3: Comparison of the adipose sequencing data with the reference genome; Table S4: Body weight of experimental chickens at different ages.

## Abbreviations

Ab	Abdominal Fat
Br	Thoracic Subcutaneous Fat
Cl	Clavicular Fat
PPARγ	Peroxisome Proliferator-Activated Receptor Gamma
CEBPα	CCAAT-Enhancer-Binding Protein Alpha
HE	Hematoxylin and Eosin
cDNA	Complementary Deoxyribonucleic Acid
RNA	Ribonucleic Acid
PCA	Principal Component Analysis
DEGs	Differentially Expressed Genes
WGCNA	Weighted Gene Co-expression Network Analysis
BP	Biological Process
CC	Cellular Component
MF	Molecular Function
KEGGs	Kyoto Encyclopedia of Genes and Genomes
GO	Gene Ontology
GS	Gene Significance
MM	Module Membership
TCA cycle	Citric Acid Cycle
ECM	Extracellular Matrix
CAMs	Cell Adhesion Molecules
