# Supplementary material for: RNA-Seq Analysis Reveals the Molecular Mechanisms Regulating the Development of Different Adipose Tissues in Broiler Chicks
Source: Animals (Basel). 2024 Mar 14;14(6):899. doi: 10.3390/ani14060899 (PMC10967598; doi:10.3390/ani14060899)
Supplement: Supplementary file 1 [file animals-14-00899-s001.zip › animals-2837247-supplementary.pdf]

Table S1. The composition and nutritional value of the basal diet (% dry matter)

**Ingredients Content%**	**Content**	**Nutrient levels (%)**	**Content**
Corn	55.87	Crude Protein	21.40
Soybean	32.05	Calcium	0.90
Mixed Oil	4.00	Total Phosphorus	0.61
Fish meal	4.2.0	Available Phosphorus	0.37
CaCO3	1.29	Total Lysine	1.30
CaHPO3	0.75	Total Threonine	0.78
Lysine	0.65	Total Methionine and Cysteine	0..62
NaCl	0.19		
Premix [1]	1.00		
Total	100.00		

Note: [1] The premix provides per kg of diets: Iron, 66 mg; Mangan, 95.4 mg; Zinc, 96.6 mg; Copper, 15 mg; Selenium, 0.41 mg; Iodine, 0.31 mg; Vitamin, 8200 IU: Vitamin D3, 1000 IU; Vitamin E, 20 IU; Vitamin B2, 10 mg; Vitamin B6, 5 mg; Vitamin B12 0.04 mg; Biotin 0.3 mg.

Table S2. Primers used for the quantitative real-time PCR (qRT-PCR).

**Gene**	**Sequence (5'-3')**
LDHA	F: TTCTCTGCCAGCTGAATAGCTT<br>R: CGGGTCATTGTCTTGTTGCAT
PPARα	F: CAGAATAAGGAAGCCGAAGT<br>R: AATGATGGCAGCGACAAA
FADS2	F: AATTGAGCACCACCTGTTCC<br>R: TGGCACATAACGACTTCACC
ACSL1	F: CAAAGGAGAAGGTGAGGTGTG<br>R: CTTCAACGTACCGTTTGGTAG
β-actin	F: TGTGCGTGACATCAAGGA<br>R: CCAGGATAGAGCCTCCAAT

F = Forward primer； R = Reverse primer.

Table S3. Comparison of the sequencing data of the adipose tissue with the reference genome.

Sample name	Total_reads	Total_map	Unique_map	Multi_map
D1_1c	40851478	90.71%	88.74%	1.97%
D1_2c	57865946	89.79%	87.89%	1.9%
D1_3c	58361972	92.14%	90.59%	1.55%
D1_1b	53362856	93.24%	91.72%	1.53%
D1_2b	60753018	93.25%	91.69%	1.56%
D1_3b	56753804	93.18%	91.63%	1.55%
D1_1a	58091868	89.09%	87.35%	1.73%
D1_2a	47074242	88.71%	86.82%	1.89%
D1_3a	54315020	88.5%	86.79%	1.71%
D4_1c	66991938	92.32%	90.73%	1.59%
D4_2c	61825580	92.6%	91.0%	1.6%
D4_3c	53538268	90.54%	89.11%	1.43%
D4_1b	56309110	90.24%	88.46%	1.79%
D4_2b	49310976	91.89%	90.51%	1.39%
D4_3b	53075198	86.2%	84.33%	1.87%
D4_1a	76983416	89.43%	87.39%	2.04%
D4_2a	49082466	89.37%	87.53%	1.84%
D4_3a	58382250	89.02%	86.44%	2.57%
D7_1c	55646882	89.92%	88.46%	1.45%
D7_2c	60952182	92.53%	91.13%	1.4%
D7_3c	61414954	91.77%	90.34%	1.43%
D7_1b	58055076	92.31%	90.88%	1.43%
D7_2b	62009242	91.41%	90.02%	1.39%
D7_3b	64544254	90.87%	89.49%	1.38%
D7_1a	64367156	91.05%	89.45%	1.6%
D7_2a	71681472	91.06%	89.64%	1.42%
D7_3a	58013170	91.41%	89.98%	1.42%
D11_1c	50069332	90.81%	89.41%	1.4%
D11_2c	59371790	92.67%	91.11%	1.56%
D11_3c	57422278	92.29%	90.74%	1.55%
D11_1b	55216378	92.56%	91.16%	1.4%
D11_2b	62105954	92.7%	91.13%	1.57%
D11_3b	50420108	92.97%	91.43%	1.55%
D11_1a	59622588	92.66%	91.07%	1.59%
D11_2a	66279870	90.78%	89.32%	1.46%
D11_3a	55835562	92.51%	90.97%	1.54%
D15_1c	61028258	92.54%	91.06%	1.48%
D15_2c	63460104	92.88%	91.3%	1.58%
D15_3c	55109590	92.51%	91.02%	1.5%
D15_1b	41426298	91.38%	89.8%	1.58%
D15_2b	68585392	92.69%	91.2%	1.49%
D15_3b	56768840	91.61%	90.15%	1.46%

D15_1a	46353958	92.77%	91.07%	1.7%
D15_2a	65877540	91.48%	89.94%	1.55%
D15_3a	55336386	93.66%	92.18%	1.49%

Note: a is abdominal fat, b is thoracic subcutaneous fat, c is collarbone fat.

Table S4. Body weight of the experimental chickens at different periods of growth.

Chicken age (days)	Body weight (g)
1	47.93
1	40.94
1	42.15
1	46
1	44.39
1	47.83
4	68.56
4	73.93
4	80.5
4	74.85
4	68.81
4	74.63
7	115.68
7	93
7	109.26
7	99.29
7	108.23
7	98.36
11	177.08
11	156.46
11	133.58
11	162.65
11	152.83
11	165.6
15	208.05
15	216.92
15	205.82
15	198
15	231.23
15	255.93

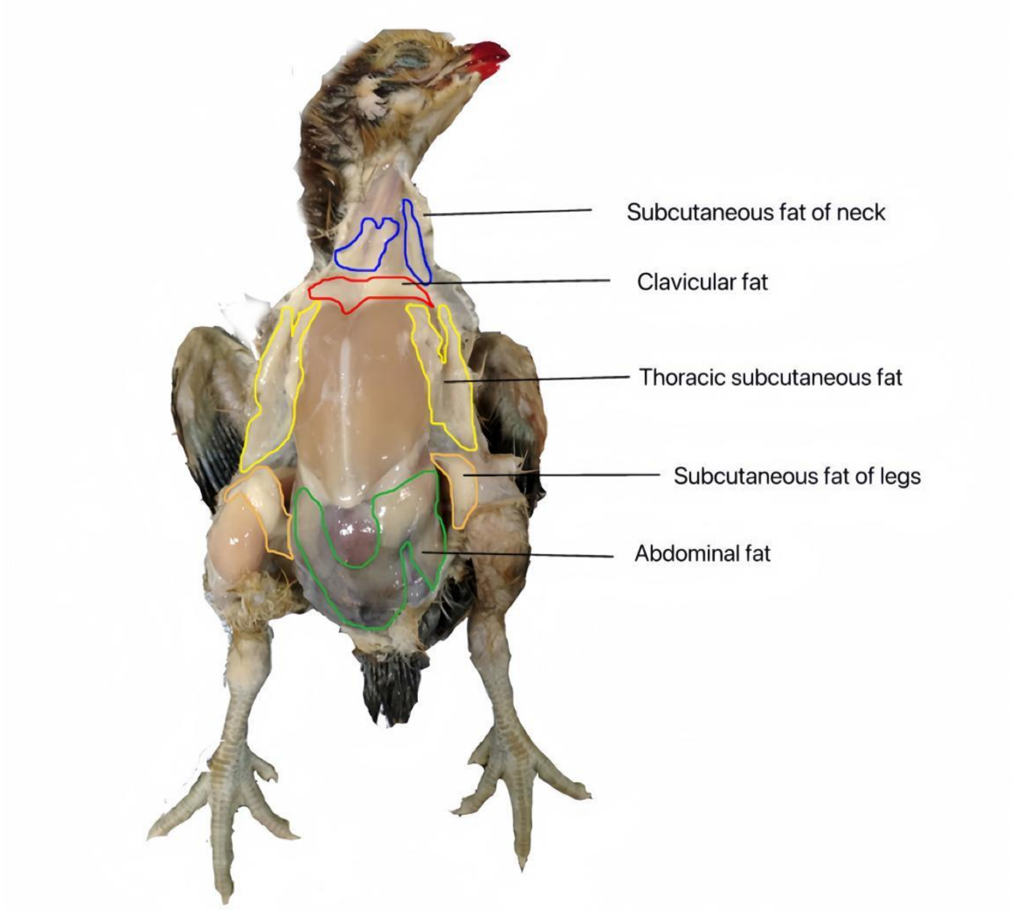

Figure S1. Adipose tissues at different body parts of the chicks.

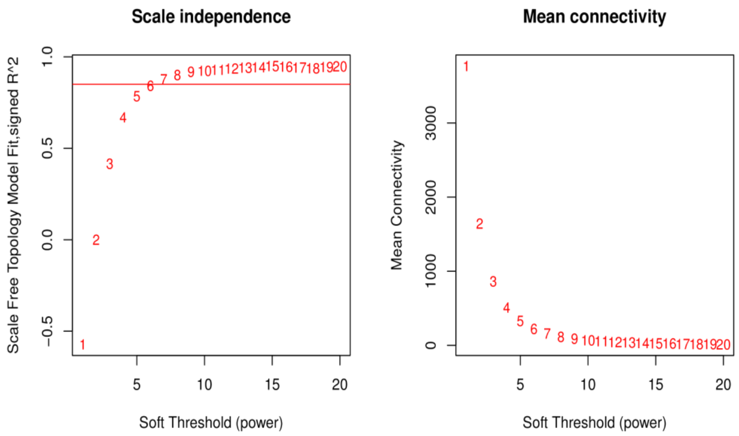

Figure S2. Selection of WGCNA soft threshold.
